# Highlighting the Potential of LyeTx I, a Peptide Derived from the Venom of the Spider *Lycosa erythrognatha*, as a Potential Prototype for the Development of a New Antimicrobial Against Carbapenem-Resistant *Klebsiella pneumoniae*

**DOI:** 10.3390/ph18050679

**Published:** 2025-05-02

**Authors:** William Gustavo Lima, Amanda Souza Félix, Felipe Rocha da Silva Santos, Fernanda de Lima Tana, Amanda Neves de Souza, Rodrigo Moreira Verly, Maria Elena de Lima

**Affiliations:** 1 Programa de Pós-Graduação Stricto Sensu em Medicina e Biomedicina, Faculdade Santa Casa de Belo Horizonte, Belo Horizonte 30150-221, MG, Brazil; 2 Departamento de Química, Instituto de Ciências Exatas, Universidade Federal dos Vales do Jequitinhonha e Mucuri, Diamantina 39100-000, MG, Brazil; amanda.felix@ufvjm.edu.br (A.S.F.); amanda.neves@ufvjm.edu.br (A.N.d.S.); verly.rodrgio@ufvjm.edu.br (R.M.V.); 3 Instituto de Ciências Biológicas, Universidade Federal de Minas Gerais, Belo Horizonte 31270-901, MG, Brazil; felipe.rocha1@live.com (F.R.d.S.S.); fernandatana@gmail.com (F.d.L.T.)

**Keywords:** carbapenem-resistant *Klebsiella pneumoniae*, superbug, antimicrobial peptides, spider venom, pneumonia, antimicrobial therapy, toxinology, pharmaceutical development, pharmacology

## Abstract

**Background**: Carbapenem-resistant *Klebsiella pneumoniae* (CRKP) is a multidrug-resistant (MDR) gram-negative bacterium frequently involved in hospital-acquired pneumonia. The infection caused by this superbug has spread quickly in health centers worldwide, leading to high mortality rates. Due to this emerging scenario, the World Health Organization has categorized CRKP as the highest-priority species for the development of new compounds. In this context, antimicrobial peptides (AMPs) stand out as prototypes for alternative antimicrobials against superbugs, including CRKP. **Objectives**: We aimed to describe the antibacterial effect of an AMP (LyeTx I), derived from the venom of the spider *Lycosa erythrognatha*, against CRKP in vitro and in a murine pneumonia model. **Results**: LyeTx I showed antibacterial effects against all the CRKP clinical isolates tested, with a minimum inhibitory concentration (MIC) range of 2–8 µM and a minimum bactericidal concentration (MBC) range of 2–16 µM. The microbial anionic membrane was the primary target of LyeTx I, which acts by displacing divalent cations bound to this structure in a manner similar to that of polymyxins. Notably, LyeTx I displayed significant lytic activity against mimetic membranes, indicating its potential to disrupt bacterial cell integrity. In in vivo assays, the LyeTx I peptide proved to be safe at a dose of 10 mg/kg. In addition, intraperitoneal use of LyeTx I reduced the bacterial load and inflammation in the lungs of animals infected with a hypervirulent strain of CRKP. **Conclusions**: These results indicate that LyeTx I is a potential prototype for the development of new antibacterials against MDR species, such as CRKP.

## 1. Introduction

Pneumonia is the most lethal infectious disease in the world, ranking as the fourth leading cause of mortality according to the World Health Organization (WHO) [1,2]. It is estimated that pneumonia kills 2.6 million people annually, exceeding the number of victims of HIV/AIDS, malaria, and tuberculosis combined [1]. Pneumonia can be septic, when caused by microorganisms, or aseptic, when it involves irritating chemical substances. Although it can be induced by different pathogens, such as viruses, fungi, and parasites, septic pneumonia caused by bacteria stands out due to its high incidence [1,3]. According to the environment where the infection is acquired, bacterial pneumonia can be categorized into community-acquired disease, caused mainly by the pathogens *Streptococcus pneumoniae*, *Mycoplasma pneumoniae*, and *Haemophilus influenzae* [4], or hospital-acquired disease, whose main etiological agents are gram-positive cocci, especially *Staphylococcus aureus*, and gram-negative bacilli such as *Klebsiella*, *Enterobacter*, *Pseudomonas*, and *Acinetobacter* [5].

*Klebsiella pneumoniae* has been recognized as the second most common gram-negative pathogen in healthcare-associated infections (HAIs), accounting for up to 10% of cases [6]. However, when we stratify the sites of infections, *K. pneumoniae* stands out as the main Enterobacteriales member associated with hospital-acquired pneumonia [7,8]. *K. pneumoniae* is a bacillary, gram-negative, encapsulated, aerobic, non-sporulating, and non-motile bacterium, with a remarkable ability to resist sanitizers and antimicrobial agents [8]. In this sense, the most significant threat is currently the growing resistance to carbapenems among strains of *K. pneumoniae* [9]. The mortality rate associated with infections caused by carbapenem-resistant *K. pneumoniae* (CRKP) can reach 40–50% [10], and a recent review revealed that more than 60% of hospitals have reported isolates with this phenotype [7]. Furthermore, outbreaks of *K. pneumoniae* strains that produce carbapenemases and are also resistant to polymyxins, the last line of therapy in these cases, have been reported in hospitals worldwide [11].

Due to the public health emergency associated with CRKP, in 2024, the WHO categorized this microorganism as a critical priority for the development of new antimicrobials, ranking it first among the 24 bacteria cited [12]. In this sense, antimicrobial peptides have shown promise as new therapeutic agents for treating infections caused by multidrug-resistant bacteria [13]. AMPs are known to have several advantages over traditional antimicrobials such as good bactericidal effects, a low potential to provoke the development of resistance, potent antibacterial activity, a lack of waste generation (due to their rapid hydrolysis in the environment), and high solubility in water [14,15]. In 2010, our research group isolated a peptide containing 25 amino acid residues, with a natural amide modification at the *C*-terminal region, whose primary sequence was defined as H-IWLTALKFLGKNLGKHLAKQQLAKL-NH_2_ (2831.1 Da). This peptide originated from the venom of the Brazilian spider *Lycosa erythrognatha*, popularly known as “tarântula”, “aranha-de-jardim”, or “aranha-lobo”, and was named LyeTx I [16]. LyeTx I exhibited high antibacterial activity against *Escherichia coli*, *Staphylococcus aureus*, *Aggregatibacter actinomycetemcomitans*, *Pseudomonas aeruginosa*, *Escherichia coli*, *Acinetobacter baumannii, Staphylococcus epidermidis*, and *Streptococcus sanguinis* [16,17,18]. Nuclear magnetic resonance studies revealed that LyeTx I is a cationic peptide that has an alpha-helix secondary structure, which allows this compound to insert itself into the anionic membrane of microorganisms, inducing cell destabilization and lysis [16]. This finding means that its action is maintained even in cells in the stationary growth phase or in cells with resistance mechanisms against conventional antimicrobials, standing out as a viable option against multidrug-resistant (MDR) bacteria [19]. However, the pharmacological effect of this peptide against *Klebsiella pneumoniae* remains to be investigated, especially in carbapenem-resistant strains. Thus, we describe the in vitro antimicrobial activity of LyeTx I against this superbug and evaluate its therapeutic potential in a model of murine pneumonia.

## 2. Results and Discussion

Firstly, the antibacterial activity of LyeTx I against carbapenem-resistant *K. pneumoniae* (CRKP) clinical isolates was evaluated by determining the minimum inhibitory concentration (MIC). As shown in Table 1, LyeTx I showed activity against all the CRKP isolates tested, with MIC range of 2–8 µM (MIC_50_ of 4 µM). Colistin, a drug known to act on CRKP, presented MIC range of 0.001–2.000 µM (MIC_50_ of 0.016 µM), thus validating our experimental conditions. According to Mbaveng et al. [20], antimicrobial activity is defined as significant when the MIC is less than 10 μM; moderate between 10 μM and 100 μM; and low when the MIC is greater than 100 μM. Accordingly, the antibacterial effect of LyeTx I was categorized as significant.

**Figure 1:**
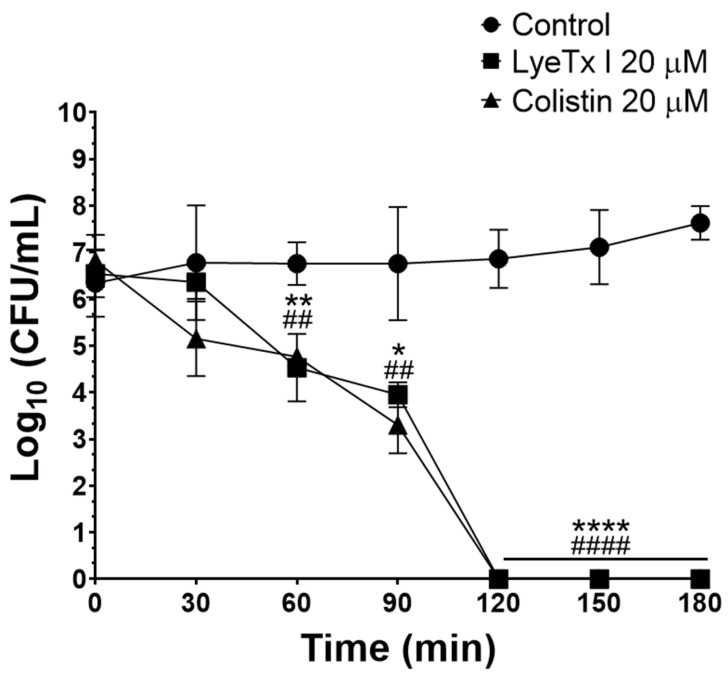
Time–kill curve of LyeTx I (20 µM; square) and colistin (20 µM; triangle) against a carbapenem-resistant *Klebsiella pneumoniae* lineage (isolate 662). The plot shows the number of logarithmic colony-forming units per milliliter (Log_10_ CFU/mL). Bacterial cells untreated were used as negative control (circle). One asterisk (*) indicates statistically different between LyeTx I and untreated cells with 0.05 < *p* ≤ 0.01. Two asterisks (**) indicate a statistical difference between LyeTx I and untreated cells with 0.01 < *p* ≤ 0.001. Four asterisks (****) indicate a statistical difference between LyeTx I and untreated cells with *p* < 0.0001. Two hashtags (^##^) indicate a statistical difference between colistin and untreated cells with 0.01 < *p* ≤ 0.001. Four hashtags (^####^) indicate a statistical difference between colistin and untreated cells with *p* < 0.0001. The results were analyzed by one-way ANOVA with Dunnett post hoc test.

Subsequently, the bactericidal potential was studied by determining the MBC. LyeTx I was able to induce bacterial death of all isolates tested within the MBC range of 2–16 μM (MBC_50_ of 4 μM), whereas the bactericidal agent used as a positive control (colistin) also killed the evaluated CRKP isolates (MBC_50_ of 4 μM) (Table 1). This result is relevant because substances with the ability to kill bacteria are strong candidates for clinical use, as they can accelerate patient recovery and reduce the chances of recrudescence of the disease [22]. Furthermore, the activity kinetics of the LyeTx I peptide and the positive control (colistin) were determined by the time–kill curve assay. LyeTx I at 20 µM was able to kill all CRKP cells (isolate 662) in the bacterial suspension tested (10^6^ CFU/mL) after 120 min of incubation, an effect that was equipotent to that of colistin (20 µM) (Figure 1). The rapid bactericidal effect demonstrated by the LyeTx I compound represents another therapeutic advantage, as the rapid removal of the infectious focus reduces the likelihood of complications of the infection, such as its dissemination or the selection of antibiotic-resistant strains during treatment [23].

**Figure 2:**
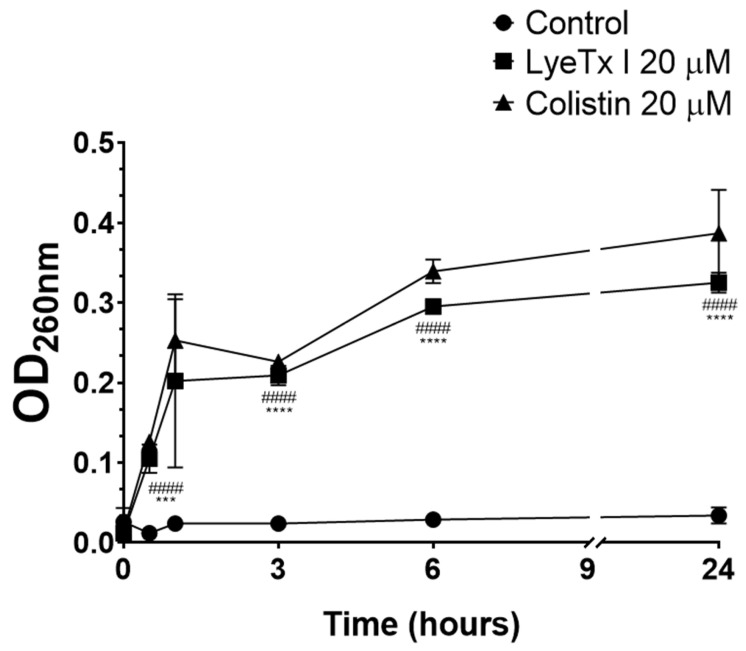
The release of ultraviolet absorbing materials (DNA/RNA) from carbapenem-resistant *Klebsiella pneumoniae* (isolate 662) was determined spectroscopically at OD_260nm_ after incubation with LyeTx I (20 µM; square) or colistin (20 µM; triangle) at several time intervals (0.5; 1; 3; 6; and 24 h). Bacterial cells untreated were used as negative control (circles). Three asterisks (***) indicate a statistical difference between LyeTx I and untreated cells with 0.001 > *p* ≥ 0.0001. Four asterisks (****) indicate a statistical difference between LyeTx I and untreated cells with *p* > 0.0001. Four hashtags (^####^) indicate a statistical difference between colistin and untreated cells with *p* > 0.0001. The results were analyzed by one-way ANOVA with Dunnett post hoc test.

The bacteriolytic effect of LyeTx I was studied by intracellular material release assay with absorbance at 260 nm (DNA/RNA). As shown in Figure 2, the peptide increased the release of intracellular material as a function of time (LyeTx I OD_260nm_ 0.325 ± 0.027 after 24 h vs. Untreated OD_260nm_ 0.034 ± 0.008 after 24 h; *p*-value < 0.0001). Colistin, an antimicrobial known to induce bacterial cell lysis, considerably increased the release of bacterial DNA/RNA (OD_260nm_ 0.411 ± 0.013 after 24 h; *p*-value < 0.0001), thus validating our experimental conditions. In general, antimicrobial peptides are known for their bacteriolytic action, which involves the disruption of the bacterial membrane [22]. Several studies using natural and synthetic lipid bilayers have clearly shown that AMPs, such as LyeTx I, interact with the lipid components of the bacterial cell membrane, promoting its destabilization and consequent disruption [22]. This phenomenon correlates with the release of cytoplasmic contents, thus justifying the increase in the concentration of products with absorbance at 260 nm (DNA/RNA) after exposure of the bacterial suspension to the peptide derived from the toxin of *L. erythrognatha*.

Polymyxins (i.e., colistin and polymyxin B) are cyclic peptides known to competitively displace outer membrane-bound divalent cations (especially Ca^2+^ and Mg^2+^), which are essential cofactors for several microbial metabolic pathways. Furthermore, membrane-bound divalent ion homeostasis contributes to membrane stabilization and osmotic resistance of the cell [24]. Therefore, to investigate whether the mechanism of bacteriolysis on CRKP (isolate 662) previously identified for LyeTx I involves displacement of divalent cations, as occurs with polymyxins, we determined the MIC of these compounds in the presence of different concentrations of CaCl_2_ or MgCl_2_. Interestingly, the activity of LyeTx I against CRKP was lost after adding Ca^2+^ and Mg^2+^ (Table 2), indicating that the peptides disrupt the membrane, at least in part, by displacing divalent ions. Colistin reduced activity in the presence of Mg^2+^ and completely lost activity with the addition of Ca^2+^ (Table 2), thus validating our experimental conditions.

**Figure 3:**
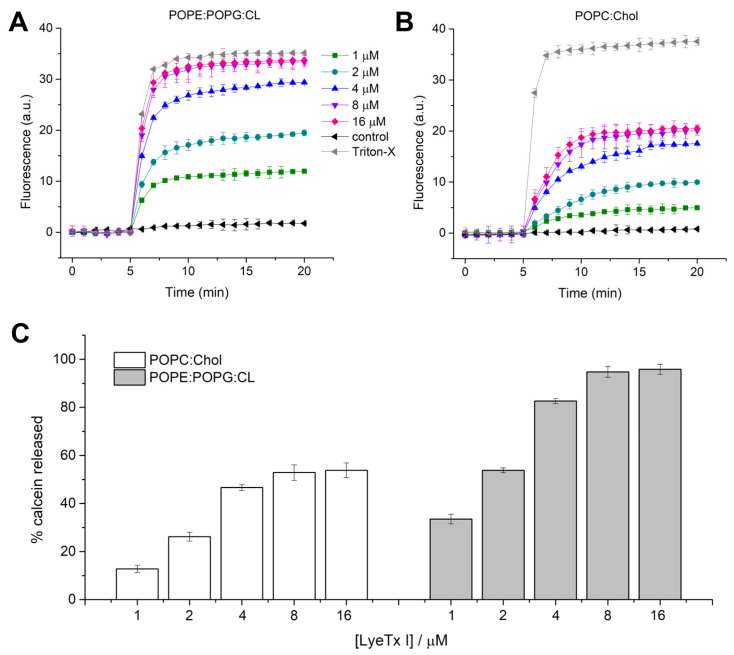
Effect of LyeTx I on synthetic phospholipid membranes as assessed by calcein release from POPE:POPG:CL (4:1:1) and POPC:Chol (3:1) LUVs, both at 100 µM lipid concentration. Effect of different LyeTx I concentrations as a function of time on POPE:POPG:CL (4:1:1) LUVs (**A**) and POPC:Chol (3:1) LUVs (**B**); percentage of calcein release relative to that observed after addition of 0.1% Triton X-100^®^ (positive control, considered as 100%) in POPE:POPG:CL (4:1:1) and POPC:Chol (3:1) LUVs treated with different concentrations of LyeTx I (**C**). Data are representative of three independent experiments.

Since LyeTx I showed a promising bacteriolytic effect, the activity of this molecule against mimetic membranes was investigated. Previous studies revealed the lytic activity and the effect of LyeTx I in anionic mimetic membranes composed of 1-palmitoyl-2-oleoyl-glycero-3-phosphocholine (POPC):1-palmitoyl-2-oleoyl-sn-glycero-3-phospho-(1′-rac-glycerol) (POPG) (3:1) [16,25]. Nevertheless, the main phospholipid composition of *K. pneumoniae* includes phosphatidylethanolamine, phosphatidylglycerol and cardiolipin (CL) [25]. Therefore, to compare the effect of the peptide on *K. pneumoniae* bacterial and eukaryotic membranes, large unilamellar vesicles (LUVs) composed of 1-palmitoyl-2-oleoyl-sn-glycero-3-phosphoethanolamine (POPE):1-palmitoyl-2-oleoyl-sn-glycero-3-phospho-(1′-rac-glycerol) (POPG):CL (4:1:1) and 1-palmitoyl-2-oleoyl-glycero-3-phosphocholine (POPC):cholesterol (Chol) were used for calcein release and differential scanning calorimetry (DSC) experiments. Calcein extravasation from both LUVs is virtually negligible in the absence of the peptide (first 5 min). LyeTx I induces calcein extravasation in LUVs formed by POPE:POPG:CL and POPC:Chol, both at a lipid concentration of 100 µM (Figure 3). An increase in fluorescence intensity was observed immediately after the addition of LyeTx I (Figure 3A,B) at all tested concentrations. The kinetics of calcein release were recorded for 15 min and the maximum fluorescence intensity was measured after 12 min of peptide addition. Calcein release from LUVs increases in both systems in a concentration-dependent manner. The maximum percentage of calcein extravasated (% calcein) from both vesicles in each peptide concentration was calculated in relation to the maximum release induced by Triton^TM^ X-100. The percentage of calcein extravasation is plotted as a function of the LyeTx I concentration in the presence of POPE:POPG:CL and POPC:Chol vesicles (Figure 3C). The lytic effect on POPE:POPG:CL LUVs was virtually similar to Triton^TM^ X-100 for peptide concentrations ≥8 µM (95 ± 3%). On the other hand, the maximum calcein % released from POPC:Chol LUVs was lower (53 ± 4%) when compared to anionic LUVs at the same LyeTx I concentration (≥8 µM), indicating that the lytic activity of the compound is greater on bacterial cells compared to mammalian cells, where cholesterol is present. This can be partially explained by the differential biophysical characteristics between bacterial and eukaryotic membranes. In fact, the former is characterized by a high density of negative charges, which provide an anchoring structure for positively charged LyeTx I. On the other hand, zwitterionic characteristics of mammalian cell membranes reduce the affinity with cationic AMPs, such as LyeTx I [14].

**Figure 4:**
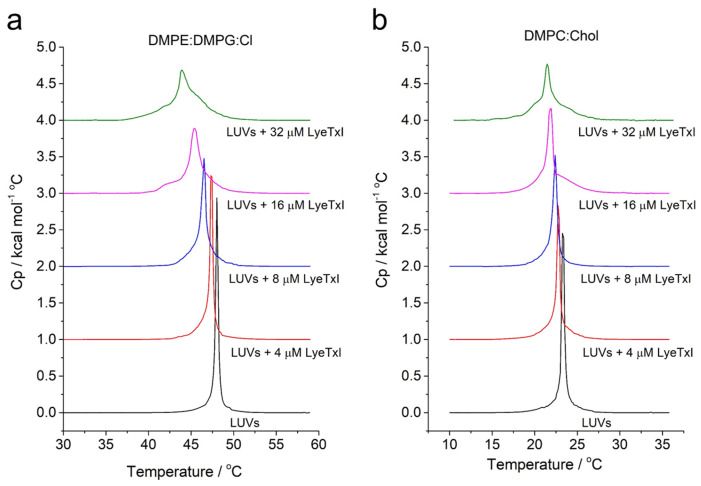
Differential scanning calorimetry (DSC) profile of the gel-to-liquid crystalline phase transition in phospholipid vesicle/LyeTx I complexes. Effects on LUVs of DMPE:DMPG:CL (4:1:1) (**a**); effects on LUVs of DMPC:Chol (3:1) (**b**).

In addition, peptide–membrane interaction on vesicles that simulate eukaryotic (i.e., DMPC:Chol (3:1, mol:mol) and bacterial membranes of *Klebsiella pneumoniae* i.e., 1,2-Dimyristoyl-sn-glycero-3-phosphoethanolamine (DMPE): 1,2-dimyristoyl-sn-glycero-3-phospho-rac-(1-glycerol) (DMPG):CL (4:1:1, mol:mol)) was also studied by DSC analysis. The effect of the LyeTx I on the stability of the membranes was evaluated by monitoring the main phase-transition temperature (*T_m_*) of phospholipid LUVs (Figure 4) [25]. Aqueous dispersions of both vesicle systems exhibit only one endothermic event, a *T_m_* near 48 °C and 22 °C for DMPE:DMPG:CL and DMPC:Chol, respectively. The DSC endothermic curves show the effect of LyeTx I on the thermotropic phase behavior of DMPE:DMPG:CL and DMPC:Chol LUVs in a concentration-dependent manner. Increasing amounts of peptide decrease the main phase transition temperature of both vesicle suspensions. Notably, the LyeTx I-induced decreases in the *T_m_* are greater in the presence of anionic vesicles (Δ*T_m_* = 4–5 °C) compared to zwitterionic ones (Δ*T_m_* = 1–2 °C). These results indicate that the presence of LyeTx I produces slight destabilization of DMPC:Chol LUVs when compared to LUVs that simulate the bacterial cell, suggesting selectivity for this compound [26].

According to the WHO, antimicrobial resistance currently represents a major public health challenge, being a multifactorial problem that requires all sectors of government and society to cooperate for effective control [6]. This phenomenon is particularly common among clinical isolates of *K. pneumoniae*, which generally show intrinsic and acquired mechanisms that encode resistance to several conventional antimicrobials [12,27]. Therefore, to analyze whether the LyeTx I induces resistance in CRKP, a multi-passage resistance selection study was performed. During the 15 days of exposure to a sub-inhibitory concentration (1/4 MIC) of LyeTx I, no resistant strains were identified, and the MIC of this peptide remained constant throughout the experimental period considered. Colistin, in turn, was also unable to induce resistance during the period evaluated. In fact, AMPs are known for their low capacity to induce resistance. This effect can be justified by their bacteriolytic action, since resistance mechanisms that involve changes in the microbial plasma membrane generally result in a reduction in the fitness or even viability of the microorganism [14].

**Figure 5:**
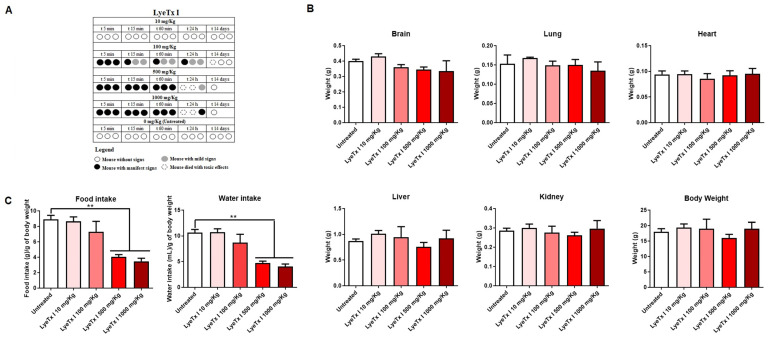
Acute toxicity of LyeTx I after intraperitoneal administration according to the OECD protocol. Lethality and clinical signs after administration of the compound (**A**). Organ mass and total body mass of the animals (**B**). Water intake and food intake of the animals (**C**). Two asterisks (**) indicate a statistical difference between LyeTx I and untreated cells with 0.01 < *p* < 0.001. The results were analyzed by one-way ANOVA with Dunnet post hoc test.

The acute toxicity of LyeTx I was evaluated in female BALB/c mice, according to the OECD protocol [28]. Animals treated with concentrations of 100, 500 and 1000 mg/kg showed evident signs of pain and discomfort immediately after intraperitoneal administration of LyeTx I, such as vocalization, abdominal curvature and manipulation of the injection area. In addition, it was possible to verify the death of two animals at the highest doses used (1000 and 500 mg/kg) and of one animal at the intermediate dose (100 mg/kg). However, no signs of discomfort or death were observed with the 10 mg/kg dose, indicating good safety at this concentration (Figure 5A), as the control group (animals administered with intraperitoneal saline) also showed no signs of discomfort or death. After euthanasia or death of the animals, the organs were weighed, as well as the total body mass. As shown in Figure 5B, there were no differences in organs and body weight between the animals that received LyeTx I and the animals in the control group (treated with saline). However, it was possible to verify that, at the highest concentrations of LyeTx I tested (1000 and 500 mg/kg), there was a significant reduction in the water and food intake of the animals (Figure 5C).

**Figure 6:**
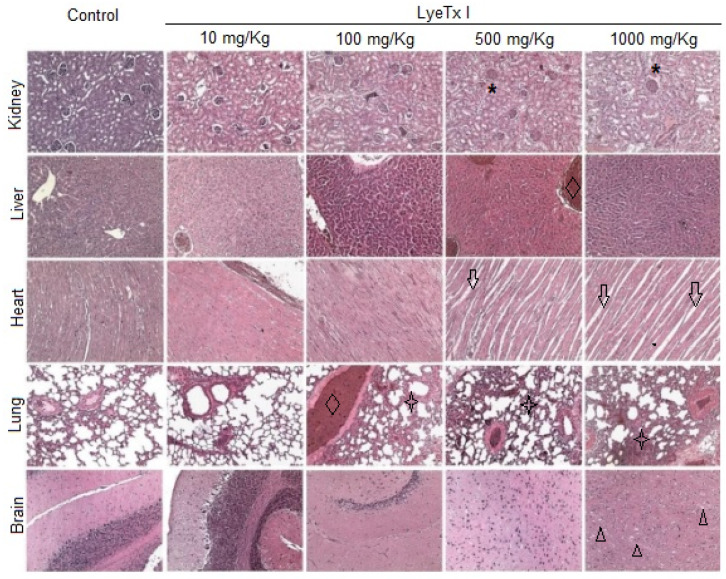
Effects of different doses of LyeTx I (10, 100, 500 and 1000 mg/kg) on histological parameters of the kidney, liver, heart, lung and brain of female BALB/c mice. In the kidney, glomerular hyperplasia can be observed, with a reduction in Bowman’s spaces (asterisk). In the groups treated with doses of 100 mg/kg or higher, an increase in hepatic basophilia was shown, suggesting the accumulation of acidic proteins, and congestion of the liver veins (diamond). The animals that received the two highest doses of LyeTx I presented atrophy of cardiac cells, as suggested by the increase in intrafibrillar spaces (black arrows). From the dose of 100 mg/kg onwards, vascular congestion and hyperemia (diamond) and an increase in the thickness of the pulmonary trabeculae (star) can be observed. Histology of the cerebral cortex reveals that only at the highest dose (1000 mg/kg) can the presence of vacuoles in tissue be detected (triangle).

Furthermore, histological analysis of the kidney, liver, lung, heart and brain was performed to verify possible micromorphological alterations in these target organs. As shown in Figure 6, the animals treated with the lowest concentration of LyeTx I (10 mg/kg) did not present any signs of renal, cardiac, pulmonary or cerebral toxicity. However, it was possible to verify a slight hydropic degeneration in the hepatocytes. At a concentration of 100 mg/kg, significant pulmonary inflammation was observed, with an increase in the thickness of the respiratory membrane, in addition to a strong hepatic basophilia, which suggests the accumulation of acidic proteins in this organ. In animals treated with a concentration of 500 mg/kg, renal damage with tubular atrophy and glomerular congestion was observed. Hepatic basophilia was also found in these animals, beyond the significant cardiomyocyte atrophy and pulmonary inflammation accompanied by edema, hyperemia and hemorrhage. Similar effects were found in animals treated with 1000 mg/kg LyeTx I, which was the only concentration at which brain changes were observed, with the presence of vacuolar degeneration in the cerebral cortex (Figure 6).

**Figure 7:**
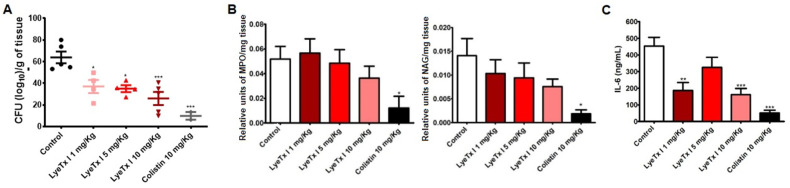
Evaluation of the efficacy of LyeTx I in a murine model of pneumonia caused by carbapenem-resistant *Klebsiella pneumoniae*. (**A**) Lung bacterial load (Log_10_CFU/g of tissue) after intraperitoneal administration of LyeTx I (1, 5, and 10 mg/kg), colistin (10 mg/kg), or saline (control) in mice (n = 5) with pneumonia induced by intranasal instillation of carbapenem-resistant *Klebsiella pneumoniae*. (**B**) Neutrophil myeloperoxidase (MPO) and *N*-acetylglucosaminidase (NAG) activity in the lung tissue of the animals. (**C**) Interleukin-6 levels in the lung tissue homogenate of animals infected and treated with LyeTx I or colistin. An asterisk (*) indicates statistical difference compared to the control with 0.05 < *p*-value < 0.01. Two asterisks (**) indicate statistical difference compared to control with 0.01 < *p*-value < 0.001. Three asterisks (***) indicate statistical difference compared to control with *p*-value < 0.0001. Results were analyzed using one-way ANOVA with Dunnett’s post hoc test.

*Klebsiella pneumoniae*, especially carbapenem-resistant strains, is one of the main pathogens involved in hospital-acquired pneumonia, and its treatment currently represents a major clinical challenge [7,8]. Therefore, with the confirmation of the potent in vitro anti-*Klebsiella* activity of LyeTx I, we aimed to investigate the efficacy of this compound in the treatment of CRKP-induced pneumonia in mice. As shown in Figure 7A, treatment of animals with 1 mg/kg (37.09 ± 6.5 Log_10_ CFU/g tissue), 5 mg/kg (35.10 ± 3.3 Log_10_CFU/g tissue) and 10 mg/kg (26.03 ± 5.9 Log_10_CFU/g tissue) was able to significantly reduce the pulmonary bacterial load compared to untreated animals (63.97 ± 5.3 Log_10_CFU/g tissue). Furthermore, it was possible to verify that colistin at 10 mg/kg significantly reduces the bacterial load (9.93 ± 3.5 Log_10_CFU/g of tissue), thus validating our experimental conditions.

One of the main complications associated with pneumonia is the intense inflammatory process formed in response to the infectious agent. This extreme event can lead to serious complications such as respiratory failure, multiple organ failure or septic shock, which usually culminate in the death of the patient [1,5]. In this sense, we aimed to investigate the effect of the LyeTx I on inflammatory parameters linked to pulmonary infection by CRKP by measuring the pro-inflammatory cytokine interleukin-6 (IL-6) and quantifying myeloperoxidase (MPO) and *N*-acetylglucosaminidase (NAG), indirect indicators of the infiltration by neutrophils and macrophages in the lung tissue, respectively. As shown in Figure 7B, treatment with the LyeTx I was not able to reduce the neutrophil or macrophage infiltrate in the lungs of animals infected with CRKP, although a slight tendency towards a reduction in the activity of both enzymes was observed. Colistin, in turn, significantly reduces the activity of these enzymes in the lungs. However, Figure 7C shows that the treatment with LyeTx I significantly reduces IL-6 levels at concentrations of 1 mg/kg and 10 mg/kg, but not at the concentration of 5 mg/kg. Moreover, colistin (10 mg/kg) also reduced IL-6 levels compared to the untreated control. These results show that LyeTx I can control the “inflammatory storm” associated with pulmonary infection by CRKP, improving the prognosis of infected patients.

Taken together, the in vitro (i.e., low MIC values, rapid bactericidal effect and bacteriolytic action at low concentrations) and in vivo (reduction in bacterial load and reduction in lung inflammation) results of the study highlight the LyeTx I as a prototype for the development of new antimicrobial agents against CRKP. However, this peptide shows some challenges that should be the target of future studies. The compound presents significant toxicity, suggesting a narrow therapeutic window. This is perhaps the greatest problem because patients with pulmonary infection by CRKP generally have low tolerance to adverse events since they are critically ill. The LyeTx I peptide has low plasma stability, being rapidly degraded by serum peptidases, which may generate the need for higher doses or an increase in the frequency of administration, generating an increased risk of adverse reactions. This limitation can be minimized with chemical modifications that reduce the access of serum enzymes to this peptide, such as modification of the N- or/and C-terminal region (e.g., *N*-acetylation and *C*-amidation), cyclization, modification of amino acids (e.g., halogenation), conjugation with macromolecules (e.g., polyethylene glycol (PEG), albumin, carbohydrate, fatty acids), and exchange of l-amino acids for d-amino acids [29]. Some species of *K. pneumoniae* with resistance to polymyxins demonstrate an alteration in charge in the outer membrane, such as the addition of positively charged moieties (phosphoethanolamine or 4-*amino*-4-deoxy-L-*arabinose)* to lipid A or loss of lipopolysaccharide, which result in the nullity of charge [30]. Since the action of the cationic peptide LyeTx I initially involves interaction with a negatively charged bacterial membrane, cross-resistance between polymyxins and the LyeTx I peptide may occur in *K. pneumoniae* and should be investigated with clinical isolates that show this phenotype.

## 3. Materials and Methods

### 3.1. Reagents

Colistin (Inlab^TM^; São Paulo, SP, Brazil), chloroform, glacial acetic acid, methanol, sodium chloride (NaCl), calcium chloride (CaCl_2_), glycine, magnesium chloride (MgCl_2_), sulfuric acid (H_2_SO_4_), sodium hydroxide (NaOH), hydrochloric acid (HCl), *Tris*-HCl, dimethyl sulfoxide (DMSO), hydrogen peroxide (H_2_O_2_), ethanol, hematoxylin, eosin (Synth^TM^; Diadema, SP, Brazil), Triton^TM^ X-100, N-acetyl-β-D-glucosamine, hexa-1,6-bis-decyltrimethylammonium bromide (HETAB), 3,3-5,5-tetramethylbenzidine, *p*-nitrophenyl-N-acetyl-β-D-glucosamine (Sigma-Aldrich^TM^; St. Louis, MO, USA), 1-palmitoyl-2-oleoyl-sn-glycero-3-phospho-(1′-rac-glycerol) (POPG), 1-palmitoyl-2-oleoyl-sn-glycero-3-phosphoethanolamine (POPE), 1-palmitoyl-2-oleoyl-glycero-3-phosphocholine (POPC), diphosphatidylglycerol (cardiolipin), 1,2-Dimyristoyl-sn-glycero-3-phosphoethanolamine (DMPE), 1,2-dimyristoyl-sn-glycero-3-phospho-rac-(1-glycerol) (DMPG), cholesterol (Chol) (Avanti Polar Lipids^TM;^ Alabaster, AL, USA), ketamine, and xylazine (Syntec^TM;^ São Paulo, SP, Brazil) were all purchased from commercial suppliers and used without further purification. Mueller–Hinton broth, Mueller–Hinton agar, and MacConkey agar were purchased from Kasvi^TM^ (São José dos Pinhais, PR, Brazil). The synthetic peptide LyeTx I was purchased from a Chinese company (Syn^TM^; Shanghai, China) and the purity was confirmed by spectrometer techniques before use.

### 3.2. Microorganisms

Twenty-one clinical isolates of CRKP that belong to the biological collection of the Faculdade Santa Casa de Misericórdia de Belo Horizonte (Belo Horizonte, MG, Brazil) were included in this study. The identification of all isolates was carried in an automated system (BD Phoenix^TM^; Treton, NJ, USA) and the resistance to carbapenems was identified by the disk-diffusion test with imipenem (10 µg), ertapenem (10 µg) and meropenem (10 µg) according to the Brazilian Committee on Antimicrobial Susceptibility Testing (BrCAST) [31].

### 3.3. Antibacterial Activity

Antibacterial activity was evaluated by determining the minimum inhibitory concentration (MIC) using the broth microdilution method according to the Clinical and Laboratory Standards Institute (CLSI) document M07 [32]. Briefly, 100 μL from a bacterial inoculum at 10^6^ colony forming units (CFU)/mL was added to sterile microplates previously filled with 100 μL of a two-fold serial dilution (1–128 µg/mL) of LyeTx I in Mueller–Hinton broth. The plates were subsequently incubated at 35 ± 2 °C for 24 h and the MIC was then defined as the lowest concentration of the peptides that completely inhibited the visible growth of microorganisms. Next, the bactericidal effect of the peptides was studied by determining the minimum bactericidal concentration (MBC) [19]. In this assay, 100 µL of the optically growth-free wells from MIC test was aliquoted and dispensed onto the Mueller–Hinton agar surface, with the MBC defined as the lowest concentration capable of inhibiting colony growth in the medium. The antimicrobial colistin was employed as a positive control.

### 3.4. Time Kill–Curve

Tubes containing an inoculum of CRKP at 10^6^ CFU/mL (isolate 662) were challenged with LyeTx I at 20 µM (10× MIC). Untreated cells and cells exposed to colistin (20 µM) were used as negative and positive controls, respectively. The tubes were incubated at 35 ± 2 °C with aeration and rotated at 225 rpm. At intervals of 0, 30, 60, 90, 120, 150, and 180 min, 100 μL of the bacterial suspension was removed from the tubes and serially diluted (10^−1^ to 10^−6^) in sterile saline (0.9% NaCl *p*/*v*). Subsequently, each dilution was plated on MacConkey agar and after incubating the plates for 24 h at 35 ± 2 °C, the bacterial load (Log_10_ CFU/mL) was determined [19].

### 3.5. Effect on the Bacterial Membrane

Release of bacterial DNA/RNA: The release of intracellular material (DNA/RNA) after treatment with LyeTx I was quantified according to Lima et al. [19], with modifications. Next, 1 mL aliquots obtained from suspensions of CRKP (10^8^ CFU/mL; isolate 662) in sterile saline were treated with LyeTx I (20 µM). The tubes were incubated at 35 ± 2 °C and at intervals of 1, 3, 6 and 24 h, 100 µL of the bacterial suspension was aliquoted and centrifuged at 1500× *g* for 25 min at 4 °C. Then, the absorbance of the supernatant at 260 nm was determined using a spectrophotometer with ultraviolet reading (Shimadzu^TM^; Tokyo, OS, Japan) and the result was expressed graphically as OD_260nm_ vs. Time (h). Colistin (20 µM) was used as a positive control and untreated cells were included as a negative control.

Displacement of divalent cations: To study the ability of LyeTx I to displace divalent cations bound to the bacterial membrane, the antimicrobial activity against CRKP (isolate 662) was determined in the presence of MgCl_2_ or CaCl_2_ [33]. Mueller–Hinton broth was supplemented with an amount of MgCl_2_ corresponding to 10, 20 and 40 µM of Mg^2+^, or with CaCl_2_ corresponding to 20, 30 and 60 µM of Ca^2+^. Finally, the supplemented medium was used to determine the MIC, been that the results were compared with the MIC in the absence of divalent cations (control). Colistin, a drug known to displace divalent cations in the bacterial membrane, was used as positive control.

Preparation of Large Unilamellar Vesicles (LUVs): The appropriate amount of phospholipid in a molar ratio of POPE:POPG:CL (4:1:1), POPC:Chol (3:1), DMPE:DMPG:CL (4:1:1) or DMPC:Chol (3:1) was transferred to a glass tube and suspended with 1 mg per mL of chloroform at room temperature. The organic solvent was removed in a rotary evaporator to form a lipid film. Multilamellar vesicles (MLVs) were prepared according to the freeze thawing method in aqueous buffer (10 mM Tris-HCl, 50 mM NaCl, pH 7.4). The resulting MLVs were subjected to eight freeze/thaw cycles using liquid nitrogen and a water bath at 35 °C (five cycles). The MLVs were extruded in a 10 mL stainless steel extruder (Lipex Biomembranes Inc.^TM^; Vancouver, VA, Canada) at 35 °C to obtain LUVs of 100 nm. For calcein release experiments, POPE:POPG:CL (4:1:1) and POPC:Chol (3:1) MLVs were prepared containing 50 mM calcein. The calcein buffer was prepared by diluting 545 mg of calcein in 2.5 mL of 1 M NaOH followed by addition of 1.25 mL of buffer (100 mM Tris-HCl, 50 mM NaCl, pH 7.4) and 2.5 mL of MilliQ^TM^ water (Millipore^TM^; Bedford, MA, USA). The solution was then pH-adjusted to 7.4 with 1 M HCl dropwise while being stirred constantly to avoid precipitation. MilliQ^TM^ water was then added to bring the calcein buffer to a final volume of 12.5 mL and calcein concentration of 50 mM. Dye-containing vesicles were separated from non-entrapped calcein using a size exclusion chromatographic column Sephadex G50 Fine (10 × 150 mm), eluted with 10 mM Tris-HCl buffer that did not contain calcein. The total lipid concentration of the extruded LUVs was estimated by a colorimetric method as described by Stewart [34].

Calcein release measurements: Calcein release experiments were carried in polystyrene microplates (128 × 86 × 14.5 mm) for fluorescence emission measurement in the presence of POPE:POPG:CL (4:1:1) and POPC:Chol (3:1) LUV suspension (100 µM) of different volumes of peptide (250 µM) and buffer (10 mM Tris-HCl, 50 mM NaCl, pH 7.4) solutions to a final volume of 300 µL as detailed in Table 3. Changes in calcein fluorescence as a function of time at 25 °C were recorded every 1 min in the Spectra Max^TM^ Paradigm detection platform (Molecular Devices^TM^; Sunnyvale, CA, USA) at excitation and emission wavelengths of 480 and 520 nm, respectively. The LUV stability was monitored for 5 min before the addition of peptide. The calcein release in the presence of the peptide was recorded for 15 min. The total calcein released from the LUVs (100%) was determined in a similar experiment using 0.1% Triton-X 100 in the absence of the peptide. Calcein leakage was calculated using Equation (1):

(1) $$Dyeleakage\left(\%\right)=\frac{\left(F-F_{0}\right)}{\left(F_{T}-F_{0}\right)}\times 100\%$$

where *F*_0_, *F* and *F_T_* denotes the basal fluorescence intensity, florescence intensity after addition of peptides and maximum florescence intensity obtained after addition of 0.1% Triton^TM^ X-100, respectively. Tris-HCl buffer was used as a negative control. The experiments were performed in triplicate on independent samples of calcein-loaded LUVs. The results are presented as the average with their standard deviations. The observed rate constant of calcein release, *k*_obs_, was calculated by using single-exponential fitting of the recorded data of fluorescence intensity as a function of time [35].

Differential scanning calorimetry: Phase transition profiles of 3 mM DMPE:DMPG:Cl (4:1:1) and 3 mM DMPC:Chol (3:1) in the absence and in the presence of LyeTx I at 4, 8, 16 and 32 µM were investigated on a VP-DSC^TM^ microcalorimeter (Malvern^TM^ Instruments, Malvern, UK). All LUV and peptide–LUV mixtures were prepared immediately before the experiments. LUV and peptide–LUV samples previously degassed were run against 10 mM pH 7.5 Tris-HCl buffer solution containing 50 mM NaCl in the reference cell. Experiments with buffers in both cells were also performed for subsequent blank correction. Three successive heating scans were performed for each sample over the temperature range of 30–60 °C for DMPE:DMPG:Cardiolipin (4:1:1) and at 10–35 °C for DMPC:Chol (3:1) at a heating rate of 1.0 °C/min. The Microcal Origin^TM^ DSC 7 (GE HealthCare-Microcal^TM^, Chicago, IL, USA) software was used for blank subtraction and transition temperature (*T_m_*—gel to liquid crystalline) determination.

### 3.6. Multi-Step Resistance Assay

The potential of the LyeTx I and control (colistin) to select resistant strains was determined by the multiple passage resistance selection study [36]. An inoculum of CRKP (isolate 662) containing about 10^8^ CFU/mL was prepared in sterile saline solution (NaCl 0.9% *w*/*v*). Next, 10 μL of this bacterial suspension was added to glass tubes containing 1 mL of Mueller–Hinton broth that was antibiotic-free (control) or supplemented with the compounds of interest at 1/4× MIC. The tubes were then incubated at 35 ± 2 °C for 24 h before each serial passage. Passages were performed for 15 consecutive days, transferring a 10 μL aliquot from the previous day’s tubes to a new tube. Before each passage, a streak of microorganism was prepared and the MIC for each streak was determined by the broth microdilution method. Finally, the MIC values of each compound were compared with cells not exposed to any antimicrobial and, in the case of a difference of at least two dilutions in the MICs between them, the induction of resistance was considered positive.

### 3.7. In Vivo Assay

Ethical issues: All experimental procedures that used mice strictly followed international protocols for the management of laboratory animals, and the methods were approved by the Ethics Committee on Research in Laboratory Animals of the Faculdade Santa Casa de Misericórdia de Belo Horizonte (protocol number: 01/2023). Before the experimental procedures, all animals were acclimatized in the experimental bioterium of the Faculdade Santa Casa de Misericórdia de Belo Horizonte for 5 days.

Toxicity in mice: The acute toxicity of the LyeTx I was analyzed in fifteen healthy female BALB/c mice according to the Organization for Economic Co-operation and Development (OECD) protocol 423 [28], with modifications. Three animals were used for each of the five peptide concentrations tested (0, 10, 100, 500 and 1000 mg/Kg), according to the recommendation of the OECD protocol 423. The animals were visually evaluated continuously in the first 60 min after intraperitoneal (i.p.) peptide administration and daily for 14 days. On the 14th day, the mice were euthanized by cervical dislocation after anesthesia (60 mg/kg ketamine + 8 mg/kg xylazine; i.p.) and the organs (brain, heart, kidney, lungs, and liver) were removed and weighed. Furthermore, water and food consumption, as well as the body weight of the animals, were evaluated throughout the experimental period. Moreover, sections of 4 µm of brain, heart, kidney, lungs and liver were prepared on a glass slide and stained with hematoxylin and eosin. The sections were subsequently analyzed under a light microscope by a pathologist (Carl Zeiss AG^TM^; Oberkochen, Germany).

CRKP-induced pneumonia model: Twenty-five six-week-old female BALB/c mice were used in this study. A model of murine pneumonia due to CRKP was conducted as described by Kumar et al. [37]. Initially, the animals were randomly divided into four groups, by means of a draw. Next, mice (*n* = 5 per group) previously anesthetized were infected by intranasal instillation with a bacterial suspension of a hypervirulent clinical isolate of CRKP (isolate 662) containing ~10^8^ CFU (40 µL). Two hours after the animals were infected, treatment began. Three groups of mice were treated with different concentrations of LyeTx I (1, 5, 10 mg/Kg; i.p. once a day) and one group received colistin as positive control (10 mg/Kg; i.p. once a day). Saline-treated mice were included as a negative control. The researcher who performed the administrations was blinded, and the treatments were identified with Arabic numerals. Moreover, the number of animals per group was determined by the sample size calculation defined by Eng et al. [38].

Determination of bacterial load: Twenty-four hours after treatments, the mice were euthanized by cervical dislocation after anesthesia and the lungs were removed aseptically. A fragment of this organ was weighed, homogenized, serially diluted in saline solution (NaCl 0.9% *w*/*v*; 10^−1^–10^−6^) and then transferred to MacConkey agar plates supplemented with meropenem (8 µg/mL). The plates were incubated at 35 ± 2 °C for 24 h and the bacterial load was then quantified (Log_10_CFU/g of lung).

Interleukin-6 quantification: Lung homogenate was centrifuged and the supernatant was collected and used to quantify interleukin (IL)-6 levels by ELISA kits (R&D Systems Inc.^TM^; Minneapolis, MN, USA), according to the manufacturer’s instructions.

Assessment of pulmonary inflammation: The presence of active infiltration in the lung by neutrophils and macrophages was assessed by the catalytic activity of the enzyme myeloperoxidase (MPO) and *N*-acetilglycosaminidase (NAG) [39]. Initially, for MPO determination, a sample of lung tissue was homogenized in phosphate buffer (PBS) and centrifuged. The precipitate was collected, diluted with 0.5% hexa-1,6-bis-decyltrimethylammonium bromide solution (HETAB; 50 mg of tissue/mL) in PBS and centrifuged (2000× *g* for 15 min at 4 °C). To 25 µL of the supernatant, 25 µL of 3,3-5,5-tetramethylbenzidine (TMB) prepared in DMSO at a final concentration of 1.6 mM was added. Subsequently, 100 µL of H_2_O_2_ was included in the system and incubated at 37 °C for 5 min. The reaction was stopped by adding 50 µL of H_2_SO_4_, and the optical density at 450 nm (OD_450nm_) was determined. Next, NAG was evaluated by dissolving the precipitate in a NaCl solution (0.9%) containing 0.1% Triton X^®^-100 and centrifuged (3000× *g* for 10 min at 4 °C). To the supernatant obtained in this step, a solution of p-nitrophenyl-N-acetyl-β-D-glucosamine diluted in sodium citrate/phosphate buffer at a concentration of 2.24 mM was added. After incubation at 37 °C for 5 min, the reaction was stopped by adding 0.2 M glycine buffer (pH 10.6) and the OD_400nm_ was determined.

### 3.8. Statistical Analysis

Data normality was assessed using the Shapiro–Wilk test. The groups were compared by one-way analysis of variance (ANOVA), using Dunnet’s post hoc test for comparison with the control group and Tukey’s post hoc test for comparison between the different doses/concentrations used. In both cases, statistical differences were considered when the *p*-value was less than 0.05. All graphs were generated using GraphPad Prism^TM^ version 5.03 software and the results were expressed as mean ± standard deviation.

## 4. Conclusions

In conclusion, the LyeTx I peptide stands out as a potential prototype for the development of new antimicrobial agents against respiratory infections caused by CRKP, a microorganism of the highest priority for the development of new drugs according to the WHO. The findings of this study demonstrate that LyeTx I exhibits a promising bacteriolytic effect against carbapenem-resistant *Klebsiella pneumoniae*, particularly through its interaction with anionic mimetic membranes. Additionally, thermotropic analysis revealed that LyeTx I selectively destabilizes bacterial membranes more than eukaryotic membranes, indicating a favorable therapeutic profile. However, it is worth noting that this compound presents considerable toxicity and, even at a dose of 10 mg/kg, which presented the best therapeutic effect, tissue alterations were observed in the liver of the animals. Therefore, chemical modifications that aim to minimize the toxicity of this compound must be performed. In addition, a formulation to increase the plasma stability of LyeTx I, or even ensure its use by inhalation, should be encouraged in order to guarantee a better pharmacokinetic profile of this compound.

## Figures and Tables

**Table 1:** Minimum inhibitory concentration (MIC) and minimum bactericidal concentration (MBC) of LyeTx I against clinical isolates of carbapenem-resistant *Klebsiella pneumoniae*.

Microorganisms	Origin	Hypermucoviscity Phenotype ^a^	LyeTx I (µM)		Colistin (µM)	
			MIC	MBC	MIC	MBC
*K. pneumoniae* 3	Respiratory secretion	Yes	4.000	4.000	0.001	0.001
*K. pneumoniae* 108	Urine	No	8.000	8.000	0.060	0.120
*K. pneumoniae* 118	Respiratory secretion	Yes	8.000	8.000	0.008	0.064
*K. pneumoniae* 168	Urine	Yes	4.000	16.000	0.063	0.500
*K. pneumoniae* 175	Urine	No	2.000	2.000	0.125	0.250
*K. pneumoniae* 200	Urine	Yes	2.000	16.000	0.016	0.063
*K. pneumoniae* 228	Hemoculture	No	2.000	2.000	0.031	0.031
*K. pneumoniae* 270	Respiratory secretion	No	4.000	4.000	0.016	0.016
*K. pneumoniae* 301	Urine	No	4.000	4.000	0.001	0.001
*K. pneumoniae* 304	Urine	Yes	4.000	4.000	0.004	0.004
*K. pneumoniae* 341	Urine	No	4.000	8.000	0.016	0.016
*K. pneumoniae* 342	Urine	No	8.000	16.000	0.002	0.002
*K. pneumoniae* 355	Respiratory secretion	Yes	4.000	4.000	0.002	0.002
*K. pneumoniae* 404	Urine	Yes	4.000	4.000	0.002	0.002
*K. pneumoniae* 422	Urine	Yes	4.000	4.000	0.063	0.500
*K. pneumoniae* 439	Urine	No	8.000	8.000	0.008	0.032
*K. pneumoniae* 599	Urine	No	4.000	8.000	0.004	0.032
*K. pneumoniae* 648	Respiratory secretion	Yes	8.000	8.000	0.060	0.060
*K. pneumoniae* 662	Respiratory secretion	No	2.000	2.000	0.500	0.500
*K. pneumoniae* 701	Urine	Yes	8.000	8.000	1.000	8.000
*K. pneumoniae* 711	Urine	Yes	4.000	4.000	2.000	16.000
MIC50			4.000		0.016	
MBC50			4.000		0.032	

^a^ The method of the International *Klebsiella pneumoniae* Study Group was adopted [21]. Colonies grown on 5% sheep blood agar were touched with a sterile wire loop and lifted vertically upward from the surface of the agar plate. Mucoid isolates adhered to the loop as it was lifted from the plate. The length of the 5 mm string of colonies was considered a positive result.

**Table 2:** Minimum inhibitory concentration (MIC; µM) of LyeTx I and colistin in the presence of divalent cations (Ca^2+^ and Mg^2+^) against a clinical isolate of carbapenem-resistant *Klebsiella pneumoniae*.

Compounds	Control	Ca^2+^			Mg^2+^		
		20 µM	30 µM	60 µM	10 µM	20 µM	40 µM
LyeTx I	2	>64	>64	>64	>64	>64	>64
Colsitin	0.5	>64	>64	>64	1	2	4

**Table 3:** Composition of samples used for calcein release measurements.

[Peptide] (µM)	V_buffer_ (µL)	V_LUVs_ (µL)	V_pep_ (µL)	Sample
0	150	150	0	1
4.2	145	150	5	2
8.3	140	150	10	3
16.7	130	150	20	4
33.3	110	150	40	5
66.7	70	150	80	6

V_pep_: Volume of peptide stock solution at 2 mM; V_LUVs_: volume of a stock solution of POPC:POPG LUVs at 200 µM; V_buffer_: volume of a Tris-Hcl buffer solution at 10 mM (pH 7.4).

## Data Availability

Data are contained within the article.
